# Waterproof-breathable PTFE nano- and Microfiber Membrane as High Efficiency PM2.5 Filter

**DOI:** 10.3390/polym11040590

**Published:** 2019-04-01

**Authors:** Xiao Li, Xiao-Xiong Wang, Tian-Tian Yue, Yuan Xu, Ming-Liang Zhao, Miao Yu, Seeram Ramakrishna, Yun-Ze Long

**Affiliations:** 1 Collaborative Innovation Center for Nanomaterials & Devices, College of Physics, Qingdao University, Qingdao 266071, China; lixiao30305@163.com (X.L.); wangxiaoxiong69@163.com (X.-X.W.); yuetiantian0427@163.com (T.-T.Y.); 13780637350@163.com (Y.X.); my2373@columbia.edu (M.Y.); seeram@nus.edu.sg (S.R.); 2 Industrial Research Institute of Nonwovens & Technical Textiles, College of Textiles & Clothing, Qingdao University, Qingdao 266071, China; zhaomingliang1978@163.com; 3 Department of Mechanical Engineering, Columbia University, New York, NY 10027, USA; 4 Center for Nanofibers & Nanotechnology, Faculty of Engineering, National University of Singapore, Singapore

**Keywords:** electrospinning, PTFE nanofiber membrane, air filter, PM

## Abstract

This study shows the feasibility of using electrospinning technique to prepare polytetrafluoroethylene/poly (vinyl alcohol) (PTFE/PVA) nanofibers on PTFE microfiber membrane as substrate. Then, PVA in the fiber membrane was removed by thermal treatment at about 350 °C. Compared to PTFE microfiber substrates, the composite PTFE fiber membranes (CPFMs) have improved filtration efficiency by 70% and water contact angle by 23°. Experimental test data showed that the water contact angle of the sample increased from about 107° to 130°, the filtration efficiency of PM2.5 increased from 44.778% to 98.905%, and the filtration efficiency of PM7.25 increased from 66.655% to 100% due to the electrospun PTFE nanofiber layer. This work demonstrates the potential of CPFMs as a filter for the production of indoor or outdoor dust removal and industrially relevant gas filtration.

## 1. Introduction

In recent years, especially in developing countries, PM2.5 pollution has become increasingly serious [1,2]. Atmospheric Particulate Matters is a general term for various solid and liquid particulate matter present in the atmosphere. According to the particle size, PM is divided into inhalable particles (PM10) and fine particles (PM2.5), which are particle sizes smaller than 10 mm and 2.5 mm, respectively [3]. Contamination of PM2.5 is very harmful because it may penetrate the human lungs and bronchi due to the small particle size [4]. Long-term exposure to PM2.5 therefore increases morbidity and mortality [5,6,7,8]. In hazy days, the concentration of PM2.5 is very high, the visibility drops drastically and the air quality is poor [3]. However, serious air pollution, especially particulate matter contamination threatens human health [9]. On account of its serious impact on public health, it is in sore need to protect the public effectively under the present hazy days. Some efforts should be made in outdoor personal protection and the protection of modern commercial buildings [10]. 

**Figure 1:**
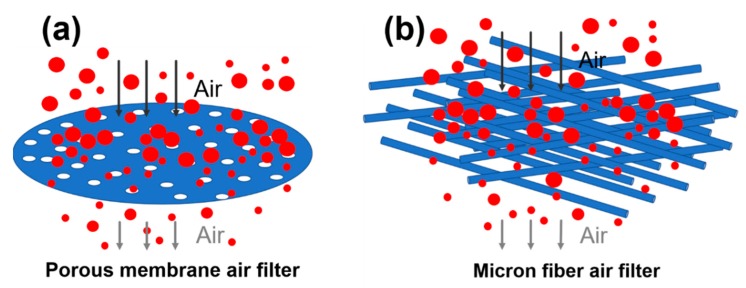
Comparison among different air filters. (**a**) Filter principle diagram of porous membrane air filter; (**b**) filter principle diagram of micron fiber air filter.

With the rapid development of industry, environmental issues have become increasingly prominent. Exhaust gas and corrosive fumes generated by industries such as thermal power generation, steel and cement are particularly serious. A common treatment method is to filter dust. The core of its technology is the high temperature filter material. Simultaneously, the real estate industry has rapidly developed. During the construction of buildings, the content of PM in the surrounding air has increased. It will not only bring harm to human health, but also cause the construction equipment to suck air with particulate matter into the suction port to aggravate the wear and damage of the machine. There are two kinds of commonly used air filters. Figure 1a shows a porous membrane filter and its filtration mechanism. It is made by forming pores on a solid substrate and in order to filter large PM it usually has a very small pore size and this type of filter has a low porosity (<30%). Therefore, despite the large pressure drop, the filtration efficiency is high. In addition, Figure 1b shows a micron fiber air filter that catches PM with a combination of a thick physical barrier and an adhesive. Filters of this type are typically made from thick fibers having a porosity of greater than 70% and multilayer diameters ranging from a few microns to or even a few tens. This type of filter is usually very thick in order to improve filtration efficiency. One disadvantage of the second filter is a compromise between filter efficiency and resistance pressure drop [3]. Shi et al. reported that the use of nanofiber filters as window screens to evaluate indoor PM2.5 from outdoor sources can reduce the average indoor PM2.5 value of outdoor PM2.5 by 65%–67% [11]. Jung et al. reported a highly efficient reduced graphene oxide filter for PM2.5 removal [12]. In this work, polytetrafluoroethylene (PTFE) is electrospun into a nanofiber membrane on the surface of a micron-sized PTFE fiber membrane, and the filtration efficiency of the micron-sized fiber membrane and CPFMs to the particulate matter is discussed. 

In recent years, electrospinning technology has received wide attention, due to its low energy consumption and as an environmentally friendly method for preparing nanofibers [13,14]. Electrospun films have properties of ultrafine fibers, high porosity, micro nanochannel interconnections, and high specific surface area [15,16,17,18,19,20,21,22,23,24]. PTFE is a thermoplastic with excellent thermal stability and chemical properties. It has a wide range of operating temperatures and can be used in the range of −190 to 260 °C. At the same time, it has lubricity, electrical insulation and aging resistance, radiation resistance [25,26,27,28] and so on. Therefore, PTFE fibers are widely used in the fields of textile industry, filter media, membrane distillation, electronic appliances and construction [29,30]. Due to its excellent chemical and physical properties, PTFE is one of the most advanced and widely used resins in fluoroplastics. It has high temperature resistance, chemical resistance and easy cleaning. It is an ideal filter material for high temperature dust filtration industry. However, due to some inherent deficiencies of PTFE, such as insoluble and ultra-high melt viscosity, it is difficult to process using conventional techniques [31,32,33,34], which may greatly limit its application. The PTFE micro-/nanofiber composite filter was prepared on the surface of PTFE microfiber membrane by coating a very thin electrospun PTFE nanofiber membrane. The purpose of this nanofiber layer is to increase the filtration efficiency and hydrophobic properties of the composite fiber membrane, thereby improve the performance of the composite membrane.

## 2. Materials and Methods

### 2.1. Materials and apparatus

The aqueous emulsion of PTFE dispersion was purchased from Zhuhai Xinying Trade Development Co., LTD., Zhuhai, China. At room temperature, a bulk density of PTFE aqueous dispersion emulsion was 1.55~1.60 g ml^−1^. The average diameter of PTFE particles in the dispersion was about 50~150 nm. The solid content of the dispersions was 60% (wt %). PVA powder (1799) was purchased from Sinopharm Chemical Reagent Co. LTD. (Shanghai, China). The PVA has a degree of polymerization of 1700 and an alcoholysis degree of 99%. The receiving end substrate used in the experiment was a microfiber PTFE membrane purchased from Shanghai Lingqiao Environment Protection Equipment Works Co., LTD (Shanghai, China). PTFE microfiber membrane is made of crystalline PTFE material, which is extruded into the film at the temperature near its melting point, and with the rapid pulling speed. After cooling, the film is extended for the second time to make it have the three-dimensional structure characteristics. All of the material was used without further purification. 

### 2.2. Nanofiber Membrane Preparation

#### 2.2.1. Solution Preparation

Firstly, the polymer and solution were weighed on an electronic scale and then stirred for several hours with a magnetic stirrer apparatus (manufacturer: Changzhou Guoyu Instrument Manufacturing Co., LTD., Changzhou, China) to obtain the homogeneous PVA spinning solution. The high concentration of PTFE aqueous emulsion and matrix polymer PVA were mixed to make a spinning solution. Finally, the polymer solution was electrospun into nanofibers at room temperature.

#### 2.2.2. Electrospinning Process

The portable electronic spinning device used for this work was PEG-1 electrostatic spinning gun, which is self-developed and manufactured by Qingdao Junada Technology Co., LTD., Qingdao, China. It has the advantages of safety, high efficiency, comfortable, flexible and so on. Its input voltage is only 220 V and the output voltage can reach 20 kV. 

**Figure 2:**
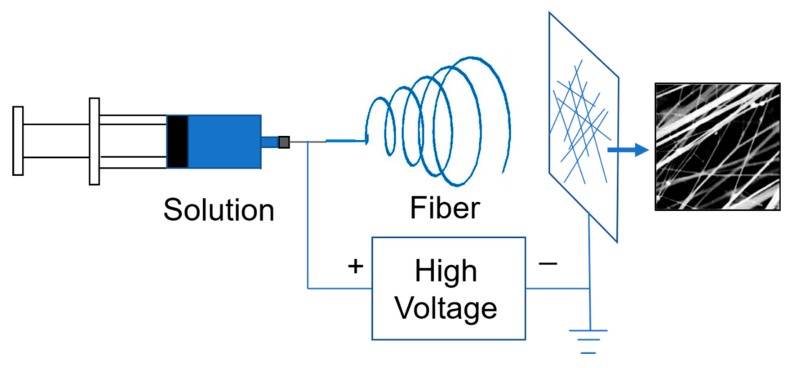
The device diagram of the electrostatic spinning process.

At 40 °C, the dispersion for electrospinning was prepared by mixing a certain amount of a PTFE dispersion and a PVA solution (12 wt %). The composition was 6 wt % PVA, PTFE: PVA = 5:1. The description was as follows: 6 wt % of PVA was related to water, and PTFE concentration was 30 wt %. Electrospinning device schematic is shown in Figure 2. Electrospinning experiments were performed at a constant applied voltage of 20 kV, an advancement rate of 0.06 mL·min-1 and a spinning distance of 11.5 cm. Figure 2 shows the device diagram of the electrostatic spinning process. Since pure PTFE is quite to be spun into nanofibers directly by solution electrospinning, a small amount of water-soluble polymer PVA was added into the PVA/PTFE dispersion as an assistant polymer. In the electrospinning process of PVA/PTFE dispersion, PVA supports the fibrous arrangement of PTFE particles. After electrospinning, the electrospun composite fibers were placed in a high-temperature oven at 350 °C for 5 min to remove PVA [30]. Finally, the hot-pressing process increases the adhesion of the composite fiber membrane. In the process of hot-pressing, the hot-pressing pressure is 1 MPa, the hot-pressing temperature is 200 °C, and the hot-pressing time is 10 s.

### 2.3. Membrane Characterization

#### 2.3.1. Characterization of e-spun Membrane

Sample microscopic morphology and diameter were characterized by SEM (Phenom Pro, Phenom-World, Eindhoven, The Netherlands). The polymer component was determined by Fourier transform infrared spectrophotometry (iS50 FT-IR, Thermo Scientific Nicolet, Shanghai, China) and Laser Micro-Raman Spectrometer (XperRam Compact, Nanobase, Shanghai, China). The pore size distribution and porosity of sample were measured using a pore size meter (PSM-165, TOPAS, Berlin, Germany). The water contact angle (WCA) measurements of each membrane sample were performed using an optical contact angle meter (JY-PHb, ChengDe JinHe Instrument Manufacturing Co., Ltd., Chengde, China). The breathability was measured by an air permeability tester (FX3300 Lab Air-IV, TEXTEST, Shanghai, China). The filter efficiency of CPFMs were investigated by a filter test bench (AFC-131, TOPAS, Berlin, Germany).

#### 2.3.2. Membrane Morphologies

The microscopic morphologies of fibrous membrane samples were researched by SEM after coating with gold. The diameters distribution of the fiber composite membrane was measured by an image analyzer (Nano Measurer, Particle Size Distribution Measurement) taken from 30 fibers. 

#### 2.3.3. Component Analysis

In order to measure the composition of the sample, infrared spectroscopy and Raman spectroscopy were separately performed on the sample. Fourier transform infrared spectrophotometry and Laser Micro-Raman Spectrometer were used to predicate the chemical composition of the samples. Raman Spectrometer uses a 532 nm laser for testing.

#### 2.3.4. Membrane Pore Size Distribution

The pore size distribution and porosity of composite plastic fiber membranes (CPFMs) were measured using a pore size meter. The instrument can measure filter papers, woven materials, non-woven and other porous materials. During the measurement, a 16 mm (red) color-coded adapters for clamping the sample. Due to the fact that PTFE is a hydrophobic material, topor aerogels were used as wetting liquids. Sample data collection and simple processing through PSMW in software, at the same time using the data as graphics software (Origin 8.5, OriginLab) for refinement.

#### 2.3.5. Water Contact Angle and Self-Cleaning

The water contact angle (WCA) measurements of each membrane sample were performed using an optical contact angle meter by the static drop method of water. The measurements were carried out at room temperature (25 ℃) and 50–60% relative humidity. Images of the droplets were collected using a lens and a light source, and the shapes of the droplets were analyzed to determine the static contact angle. The static contact angles were recorded separately and the average was calculated. 

First, a layer of visible dust is scattered on the sample, and then the sample is tilted at a certain angle. Finally, a dropper is dropped on the surface of the nanofiber membrane to observe whether the water droplet can be carried away during the downward rolling process. This is the test of self-cleaning ability of this experiment.

#### 2.3.6. Membrane Breathability Test

The breathability of CPFMs was determined by an air permeability tester, which is determined from the membrane thickness of CPFMs. When the test sample is clamped on the test head, the suction pump will start automatically. Therefore, the test pressure can only be set after the test sample is clamped. The pre-selected test pressure can be set automatically and remain unchanged. The measurement pressure was set to 200 Pa and the test area is 20 cm^2^. After a few seconds, the air permeability of the test sample was displayed and recorded according to the pre-selected measurement unit.

#### 2.3.7. Membrane Filter Efficiency

The filter efficiency of CPFMs were investigated by a filter test bench. The instrument uses a particle counting method to measure the filtration efficiency of the fabric, and it has a portion for leak detection and dust generation. It is a particle-forming agent provided to aerosol generators to produce suspended particles. During the test, the used aerogel was Dioctyl sebacate (DEHS aerogel). The control and data acquisition software (PAFWin) controls the test bench’s situation and the results of the test data recording.

#### 2.3.8. Reproducibility Test

During the experiment, we selected three samples with similar breathability to test the ventilation rate and removal efficiency 10 cycles test, calculate the standard deviation, and draw the error strip image.

## 3. Results

### 3.1. Micromorphology Analysis

In order to confirm that the prepared composite PTFE fiber membranes are composed of a micron-sized polyester substrate and nano-sized PTFE, we performed a SEM test on the sample. 

**Figure 3:**
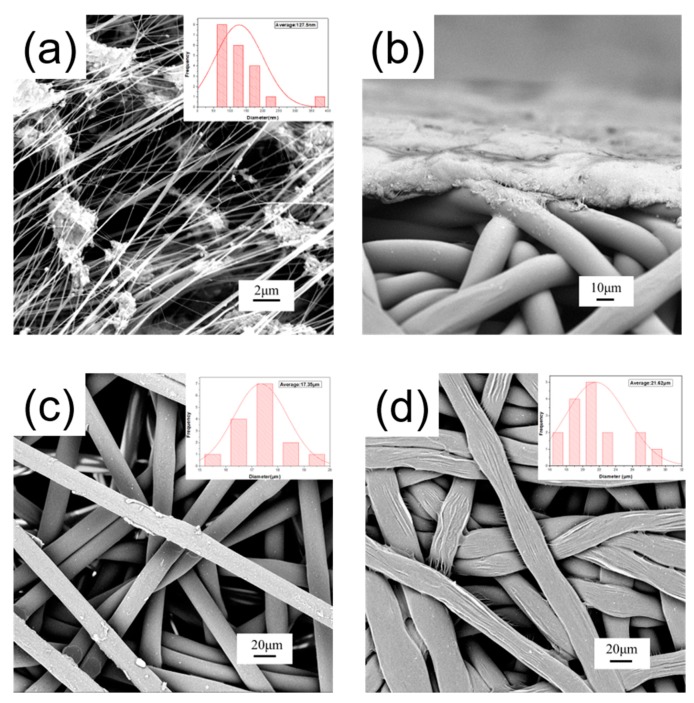
SEM images of electrospun CPFMs. (**a**) Nanofiber surface; (**b**) CPFMs cross profile; (**c**) micron substrate (without hot pressing); (**d**) micron substrate (after hot pressing).

Figure 3 shows the SEM image of the surface and substrate of the composite PTFE fiber membranes, along with its cross section (Figure 3b). From the SEM image of the sample, it can be seen that the average diameter of the fiber on the surface of the composite plastic filter membranes is 127.5 nm (Figure 3a). Figure 3b show the SEM image of the CPFMs cross section at a magnification of 1500×. It is evident from the cross-sectional SEM image of the sample that the CPFMs are compounded by two layers of fiber membranes. Figure 3c,d show the SEM image of the substrate at a magnification of 1000×, which is including hot pressing structure (Figure 3c) and no hot-pressing structure (Figure 3d). By comparing and measuring the fiber diameters before and after the hot pressing, it can be clearly seen that the fiber diameter after hot pressing increases, and the bonding between the fibers is better. The average fiber diameter of the hot-pressed part of the substrate is 17.35 μm (Figure 3c), and the fiber diameter increases to 21.62 μm (Figure 3d) after hot pressing. 

According with the above SEM images, the CPFMs is made by a thin layer of electrospun PTFE nanofiber membrane on the PTFE microfiber substrate.

### 3.2. Component Analysis

**Figure 4:**
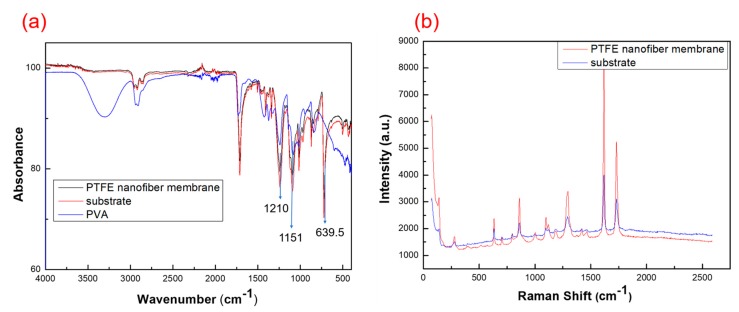
Composition comparison of PTFE nanofiber membrane and substrate. (**a**) Infrared spectroscopy image; (**b**) Raman spectroscopy image.

In order to identify the composition of the nanofiber membrane and the microfiber membrane, we performed infrared spectroscopy and Raman spectroscopy on the samples. By comparing the infrared spectrum of the nanofiber membrane and the substrate, it is apparent that the composition of the two components is the same (as shown in Figure 4a). We observed strong absorption peaks at 1210 cm^−1^ and 1151 cm^−1^, which correspond to the asymmetric stretching peaks and symmetric stretching peaks of –CF2 in the PTFE molecular structure. As shown in Figure 4b, the red and blue colors in the figure are the Raman spectra corresponding to the PTFE nanofiber film and the substrate, respectively. By comparison, it can be seen that the two are the same material. In summary, the nanofiber film we prepared is the same as the material composition of the substrate, and the material is PTFE.

### 3.3. Membrane Pore Size Distribution

**Figure 5:**
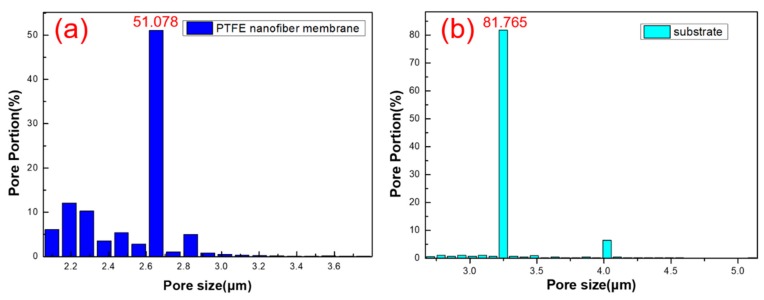
Image of membrane pore size distribution and porosity. (**a**) PTFE nanofiber membrane; (**b**) membrane substrate.

The study found that the pore size of the fiber membrane will affect the ability of air filters to filter particulate matter. If the pore size is too large, the air resistance of the fibrous membrane is small, but the filtration performance is poor. Similarly, the pore size of fiber membrane is too small, which leads to an increase in filtration and increases the resistance to air. Pore size measurements were performed on the front and back of the CPFMs, respectively. Figure 5 shows the distribution of the pore size. The average pore size of the PTFE nanofiber membrane is 2.561 μm, and pore portion is 51.078%, as shown in Figure 5a. Figure 5b shows that the average pore size of the substrate is 3.249 μm which is about 81.765%.

In the case of classical filtration theory, the stable stage of the fiber filtration can be further categorized into the following five trapping mechanisms: Interception, inertial impaction, Brownian diffusion, the electrostatic effect and the gravity effect. During the filtration process, the movement of the aerosol particles typically deviates from the gas flow, especially as they approach the fiber. Huang et al. [35] discussed the above five mechanisms in detail, because the motion deviation greatly affects the filtration performance of the membrane, and studies on the filtration mechanism of particulate matter will be useful. In addition, the electrostatic effect also firmly attaches the particles to the surface of the fibers. In addition to interception, the CPFMs we have prepared for PM capture mechanisms, such as inertial impact, Braun diffusion and electrostatic effects. This also explains why the sample we prepared has a pore size >2.5 μm, but the filtration effect on PM2.5 is still very good.

From the SEM images, it can be seen that the PTFE nanofiber membrane structure is relatively bulky and the structure of the micron substrate is relatively dense. Due to the fact that CPFMs are a kind of composite membrane material, their structure is crisscross and intertwined, so the membrane pore size is a factor that can’t be ignored. The combination of nanoscale surface and microscale substrate will not only increase the filtration resistance, but also improve the filtration efficiency.

### 3.4. Hydrophobic Characterization

**Figure 6:**
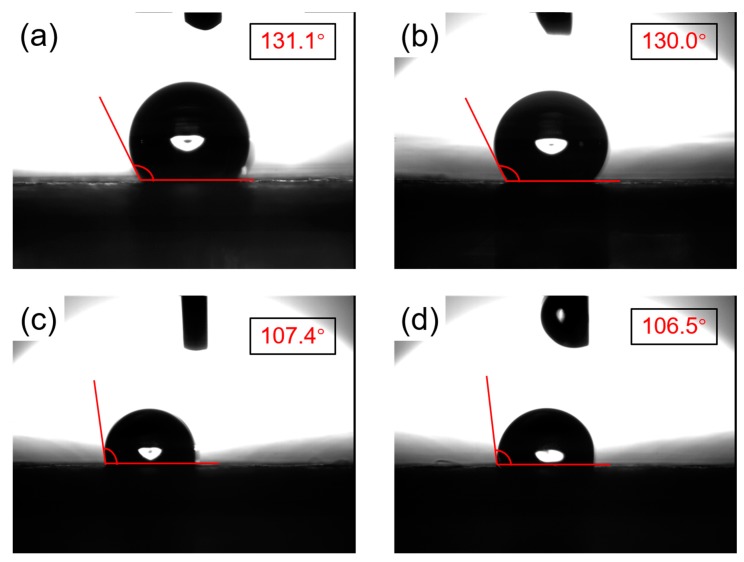
The optical image of WCA. (**a**,**b**) Contact angle of the PTFE nanofiber membrane is 131.1° and 130.0°; (**c**,**d**) contact angle of the PTFE microfiber membrane substrate is 107.4° and 106.5°.

As a waterproof-breathable nano- and microfiber membrane, it is first required to have good waterproof properties. Figure 6 shows that the CPFMs exhibited strong hydrophobicity. The WCA of the PTFE nanofiber surface is measured to be approximately 130°. Two sets of data in the measurement data are selected, which are 131.1° (Figure 6a) and 130.0° (Figure 6b), respectively. At the same time, the micron-sized substrate also has hydrophobic properties, with measured WCA of 107.4° (Figure 6c) and 106.5° (Figure 6d), respectively. Although both are hydrophobic, it is apparent that after electrospun a layer of PTFE nanofiber membrane, the hydrophobic properties are greatly improved, and the PTFE nanofiber membrane is more hydrophobic than the microfiber membrane substrate. The WCA of the PTFE nanofiber membrane is large, and it is a partial wetting relationship with water. Compared with most plastics, the hydrophobic property is very good.

To be more specific, the WCA of the surface of the PTFE nanofiber membrane prepared by the electrospinning method is larger than that of the PTFE microfiber membrane substrate and has better hydrophobic properties. However, a water droplet was stably supported on the hierarchical structure of fibrous membrane surface and air could form air pockets in the interface. This is the major reason that the CPFMs could realize hydrophobicity. 

**Figure 7:**
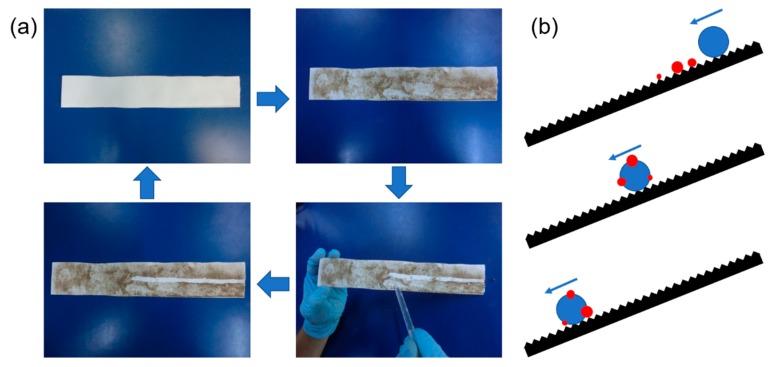
Self-cleaning ability. (**a**) Self-cleaning experiment process picture; (**b**) self-cleaning schematic.

The surface tension of PTFE is as small as 0.019 N/m. Therefore, PTFE has good non-stick properties. Therefore, when the particulate matter accumulates on the surface of the PTFE nanofiber membrane, it is easy to clean. Due to its good hydrophobic properties, it can be directly cleaned with water. Due to the many excellent properties of PTFE, the prepared PTFE nanofiber membrane has a very good self-cleaning ability, and Figure 7a shows the ability of the fiber membrane to clean dust. Figure 7b is a self-cleaning schematic diagram in which the red dots are PM and the blue dots are water droplets. During the process of water droplets falling off the surface of the fiber membrane, the water droplets carry away the PM.

### 3.5. Membrane Breathability Test

**Figure 8:**
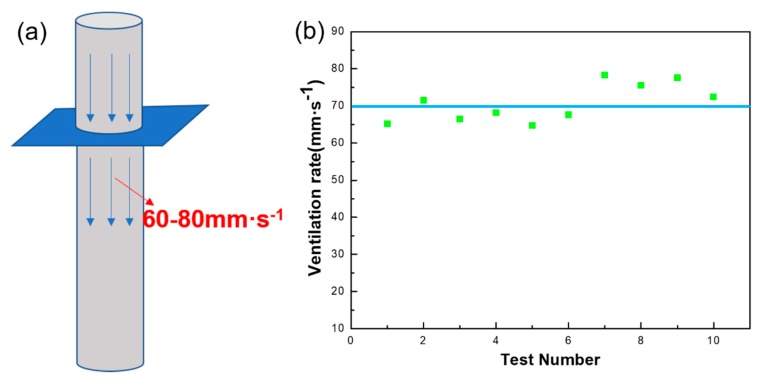
The air permeability of CPFMs. (**a**) Schematic diagram of air permeability tester; (**b**) ventilation rate of the CPFMs, which is between 60 to 80 mm·s^−1^.

Many studies have found that breathability is one of the important parameters of the membrane filter cartridge and will directly affect the filtration efficiency of the membrane filter cartridge. The breathability of the CPFMs was measured several times using a permeability tester and the data recorded. Figure 8a shows that the schematic diagram of air permeability tester. At the same time, 10 sets of data were selected for processing. As shown in Figure 8b, the ventilation rate of 10 data about the CPFMs. The blue line in the picture is guide to the eyes, and the green one is the test point. Obviously, the air permeability of the sample is approximately 70 mm·s^−1^, which explains why CPFMs have better air permeability. Similarly, poor air permeability will lead to increased pressure drop across the CPFMs, which affect the efficiency of filtration. 

Air permeability is an important parameter of the membrane. Liang et al. [36] prepared a superhydrophobic self-cleaning beaded SiO_2_@PTFE nanofiber membrane for waterproof and breathable applications. The permeability of this nanofiber membrane is only 7.2 mm·s^−1^, and the permeability of the PTFE composite fiber membrane we developed is about 70 mm·s^−1^. The permeability of PTFE film produced by Shanghai Ling Fluor Film Technology Co., Ltd., (Shanghai, China) is 64.26 mm·s^−1^. Lin et al. [37] prepared a PTFE fabric for use in high temperature filtration garments. The fabric has good gas permeability, the maximum gas permeability can reach 21.50 cc/cm^2^/s (215 mm·s^−1^), and the smallest can reach 8.11 cc/cm^2^/s (81.1 mm·s^−1^). This fabric has good gas permeability, but its filtration efficiency for PM has not been reported.

### 3.6. Filtering Performance

Membrane filtration technology has a very high dust collection capacity and therefore has an important significance in the field of air filtration. Dust holding capacity and filtration efficiency are one of the most interesting properties of the filter material characteristics, which is determined by the pressure difference upstream and downstream of the filter material to be measured and the particle size of the test particle.

**Figure 9:**
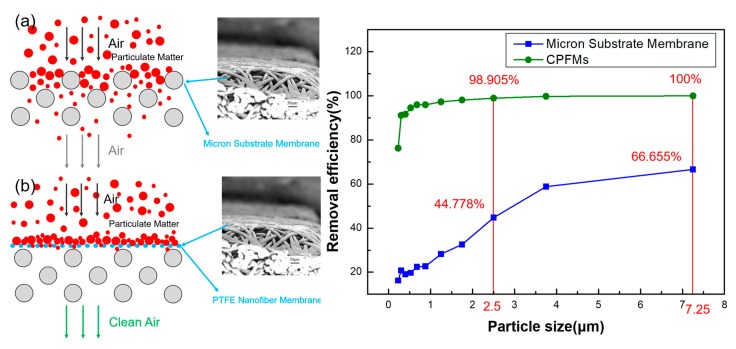
CPFMs used in air filtration to removal particulate matter and the measured removal efficiency. (**a**) Schematic diagram of micron substrate membrane filter. (**b**) Schematic diagram of CPFMs filter. (**c**) The removal efficiency of micron substrate membrane and CPFMs for different particle sizes.

Figure 9a shows the filter schematic drawing of micron substrate membrane filtration Particulate Matter (PM). As shown in Figure 9b, the filter schematic drawing of CPFMs filtration PM. According to Figure 9c, it can be seen that the filtration efficiency of the micron-sized substrate film is only 44.778% for the particle material with a particle size of 2.5 μm. After the PTFE nanofiber membrane is electrospun on the substrate by electrospinning, the filtration efficiency increases to 98.905%. The filtration efficiency for particulate matter with a particle size of 7.25 μm also increased from 66.655% to 100% (Figure 9c). It is obvious from the figure that when air containing PM passes through CPFMs, it becomes clean air. PTFE nanofiber membrane is very important for the filtration of PM, and micron-sized substrates as supporting materials increase the mechanical strength of CPFMs. 

### 3.7. Reproducibility Characterization

**Figure 10:**
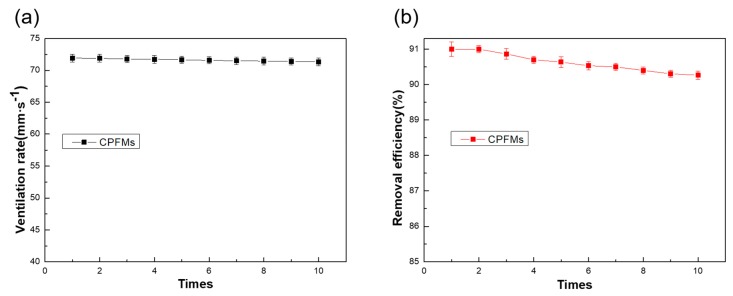
Reproducibility of the ventilation rate and removal efficiency of the composite fiber membrane. (**a**) Reproducibility of the ventilation rate. (**b**) Reproducibility of the removal efficiency.

The recycling of composite fiber membranes is also an important property. Figure 10 shows the reproducibility of the ventilation rate and removal efficiency of the composite fiber membrane. An error bars of the ventilation rate for 10 cycles of testing is shown in Figure 10a. It can be seen that after 10 cycles times of filtration test, the air permeability is not significantly reduced, and still has good air permeability. Figure 10b shows the error bars for the comprehensive removal efficiency of the 10 cycles of the filtered test sample. The initial comprehensive removal efficiency of the sample was 91%. After 10 cycles, the PM removal efficiency of the composite fiber membrane was reduced by 0.6%, and the filtration performance was still excellent.

## 4. Discussion

The composite filter is fully made of PTFE, which is a very good polymer. However, the filter will only inherit the character of PTFE. In recent years, nanocomposite-based polymer blends for membranes have become increasingly important in various fields, such as PBI/PIM blends [38], PVDF-HFP/PVDF blends [39], PBI/PDA blends [40], PEI/PVA blends [41] and PSf/sPAEs blends [42] exhibited good performance. So compositing PTFE with other materials still needs further investigation. Meanwhile, some chemical modification may further extend the performance of the fiber [43,44]. Such exploration will greatly enhance the performance of the polymer filter.

## 5. Conclusions

In summary, we have fabricated a composite PTFE filter through a portable electrospinning device. Compared with the traditional PTFE microfiber filter membrane, it not only has good hydrophobicity and gas permeability, but also has a significant improvement in filtration effect. By measurement, the water contact angle of the sample increased from about 107° to about 130°, the filtration efficiency of PM2.5 increased from 44.778% to 98.905%, and the filtration efficiency of PM7.25 increased from 66.655% to 100%. The measured gas permeability was on a scale of 60–80 mm s^−1^ in the 200 Pa. Electrospinning a thin layer of PTFE nanofiber membrane on a PTFE microfiber substrate can improve the filtration and water resistance of the microfiber membrane. These results indicate that the composite PTFE filter membrane prepared by us has the advantages of good water repellency, good gas permeability and high filtration performance, and can be applied as a filter material in many fields.

## Abbreviations

PTFE	polytetrafluoroethylene
PVA	poly (vinyl alcohol)
CPFMs	composite PTFE fiber membranes
PM	particulate matter
